# The Association between Urine *N*-Glycome and Prognosis after Initial Therapy for Primary Prostate Cancer

**DOI:** 10.3390/biomedicines12051039

**Published:** 2024-05-08

**Authors:** Tijl Vermassen, Nicolaas Lumen, Charles Van Praet, Nico Callewaert, Joris Delanghe, Sylvie Rottey

**Affiliations:** 1 Department Medical Oncology, Ghent University Hospital, 9000 Ghent, Belgium; 2 Biomarkers in Cancer, Department Basic and Applied Medicine, Ghent University, 9000 Ghent, Belgium; 3 Cancer Research Institute Ghent, 9000 Ghent, Belgium; 4 Department Urology (ERN eUROGEN Accredited Centre), Ghent University Hospital, 9000 Ghent, Belgium; 5 Uro-Oncology Research Group, Department Human Structure and Repair, Ghent University, 9000 Ghent, Belgium; 6 Department Molecular Biomedical Research, VIB-UGent Center for Medical Biotechnology, 9052 Ghent, Belgium; 7 Department Biochemistry and Microbiology, Ghent University, 9000 Ghent, Belgium; 8 Department Diagnostic Sciences, Faculty of Medicine and Health Sciences, Ghent University, 9000 Ghent, Belgium; 9 Drug Research Unit Ghent, Ghent University Hospital, 9000 Ghent, Belgium

**Keywords:** *N*-glycosylation, urine, prostate cancer, active surveillance, prognosis, prostatectomy, radiotherapy

## Abstract

Next to prostate-specific antigen, no biochemical biomarkers have been implemented to guide patient follow-up after primary therapy for localized prostate cancer (PCa). We evaluated the prognostic potential of urine *N*-glycome in terms of event-free survival (EFS) in patients undergoing primary therapy for PCa. The prognostic features of the urine *N*-glycosylation profile at diagnosis, assessed in 77 PCa patients, were determined in terms of EFS next to standard clinical parameters. The majority of patients were diagnosed with International Society of Urological Pathology grade ≤ 3 (82%) T1–2 tumors (79%) and without pelvic lymph node invasion (96%). The patients underwent active surveillance (14%), robot-assisted laparoscopic prostatectomy (48%), or external beam radiotherapy (37%). Decreased ratios of biantennary core-fucosylation were noted in patients who developed an event, which was linked to a shorter EFS in both the intention-to-treat cohort and all subcohort analyses. Combining the urine *N*-glycan biomarker with the D’Amico Risk Classification for PCa resulted in an improved nomogram for patient classification after primary therapy. The rate of urine *N*-glycan biantennary core-fucosylation, typically linked to more aggressive disease status, is lower in patients who eventually developed an event following primary therapy and subsequently in patients with a worse EFS. The combination of urine *N*-glycan biomarkers together with clinical parameters could, therefore, improve the post-therapy follow-up of patients with PCa.

## 1. Introduction

Prostate cancer (PCa) is the most common cancer in European men. The risk of dying of PCa before the age of 75 is estimated to be 1% [1]. Diagnosis is based on the outcome of several clinical parameters, such as measurements of serum prostate-specific antigen (sPSA), digital rectal examination (DRE), transrectal ultrasound, multiparametric magnetic resonance imaging, and histopathological evaluation [2,3].

In cases of localized PCa, different therapeutic options can be offered to the patients, depending on the extensiveness of the disease status.

Active surveillance (AS) allows for the close monitoring of patients with low-grade, asymptomatic PCa by means of sPSA measurements, MRI scanning, and repeated biopsies until the time that the disease evolves from an indolent to a more aggressive status [2,4]. For patients with higher-grade local PCa, radical prostatectomy (RP) can be offered. During RP, the entire prostate and seminal vesicles are removed, whether or not in combination with a pelvic lymph node dissection (PLND) [5,6]. Otherwise, primary external beam radiotherapy (EBRT) with or without androgen deprivation therapy (ADT) is a third standard treatment option for local/localized intermediate- or high-risk PCa. During radiotherapeutic treatment, the prostate and surrounding prostate bed are irradiated using intensity-modulated radiotherapy or volumetric arc radiotherapy [7,8]. The use of RP and EBRT results in a significant overall survival benefit, improved biochemical event-free survival (EFS), and reduced local, regional, and distant failure [2,9,10,11].

However, during surveillance or following primary therapy, a number of patients will experience biochemical recurrence (BCR) or progressive disease (PD). In patients surveyed using AS, approximately 27% will experience tumor upgrading, increasing to International Society of Urological Pathology (ISUP) grade ≥ 2 PCa [2,12,13]. Moreover, rising sPSA is seen in 27% to 53% of patients undergoing RP or receiving EBRT [2,14,15]. Following RP, biochemical recurrence (BCR) manifests itself as increased sPSA concentrations, which will trigger referral for salvage therapy in the case of two consecutive sPSA increases above ≥0.2 ng/mL [16]. For EBRT, BCR is defined as an increase of 2 ng/mL or more above the nadir PSA [2,17,18]. In order to improve risk classification, the European Association of Urology (EAU) has established risk groups for BCR. This classification is based on T stage, N stage, initial sPSA (iPSA), and ISUP grading. Nevertheless, D’Amico Risk Classification is not clinically implemented when guiding the post-therapeutic management of PCa patients [2].

Only a limited number of tissue-based biomarkers (e.g., Decipher^®^ [GenomeDx Biosciences, San Diego, CA, USA]) have emerged that impact clinical decision taking during patient follow-up [19,20]. Moreover, no biochemical markers, other than PSA, are available to guide patient follow-up after primary therapy.

Glycomics could, therefore, be an asset. Glycosylation, in particular asparagine- (*N*-) linked glycosylation, is a post-translational modification that results in the formation of carbohydrate structures at the *N-X-S/T* amino acid sequence of glycoproteins. These carbohydrates consist of complex structures, namely a core carrying a multitude of outer antennae, ranging between two (biantennary) or more antennae (mostly triantennary and tetraantennary). *N*-linked glycosylation is strongly biochemically influenced and can be altered in cancer. Our research group has previously demonstrated that aberrations in the *N*-glycome show tremendous potential as diagnostic and prognostic biomarkers in PCa [21,22,23,24]. However, no data exist on the prognostic properties of urine *N*-glycans following primary PCa therapy. As this is an untouched source of information, the objective of this study is to investigate the possibility of PCa patient prognostication after primary therapy based on the urine *N*-glycan patterns observed at diagnosis.

## 2. Materials and Methods

### 2.1. PCa Patients

**Figure 1:**
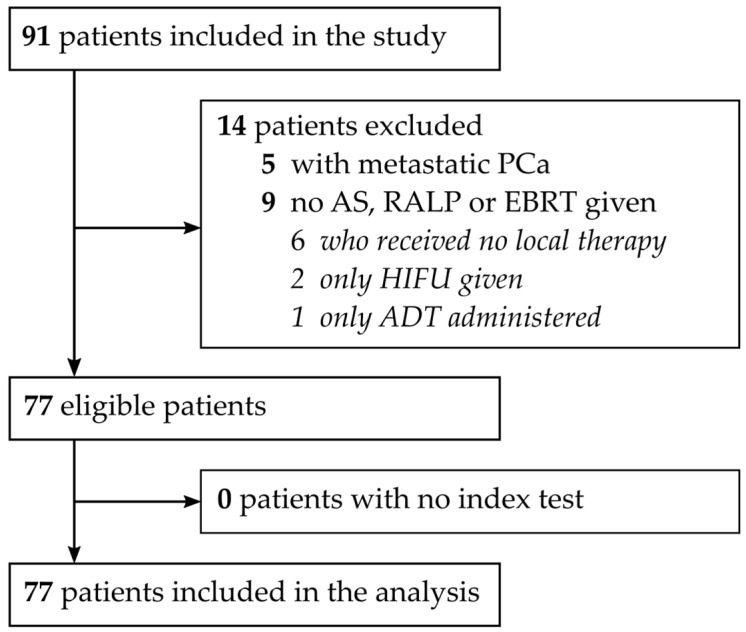
STARD flowchart diagram of PCa patients included: ADT, androgen deprivation therapy; AS, active surveillance; EBRT, external beam radiotherapy; HIFU, high-intensity focused ultrasound; PCa, prostate cancer; RP, radical prostatectomy; and STARD, Standard for Reporting Diagnostic Accuracy.

From July 2012 to November 2015, a monocentric cohort of PCa patients (*n* = 91) presenting at the Department of Urology at the Ghent University Hospital for disease diagnosis were recruited and prospectively analyzed. One single spot urine sample was collected directly following DRE at the moment of diagnosis. All relevant clinical information (initial sPSA, clinical and pathological assessment, therapy administered, and patient follow-up) was retrieved from electronic patient records. Tumoral (T) stage was determined using the following sequence of availability: (1) pathological assessment (only for patients who underwent RP), (2) imaging on MRI, or (3) through clinical evaluation (DRE). Comparably, the nodal (N) stage was assessed using (1) a pathological assessment or (2) imaging via MRI. Following exclusion (metastatic disease [*n* = 5] or no AS, RP or EBRT given [*n* = 9]), a total of 77 patients were included in the final analysis. The Standard for Reporting Diagnostic Accuracy flow diagram is presented in Figure 1.

The study was approved by the Ghent University Hospital Ethics Committee (Belgian registration number: B670201214356), and signed informed consent documentation was obtained from all patients.

### 2.2. Urine Total N-Glycome

The determination of the *N*-glycosylation profile of the urinary prostate proteins, contained in 500 microliter of urine, was carried out according to the on-membrane deglycosylation method. In short, released *N*-glycans were labeled using 8-aminopyrene-1,3,6-trisulphonic acid (Molecular Probes, Eugene, OR, USA) and desialylated using *Arthrobacter ureafaciens* α-2,3/6/8-sialidase. Two microliters of desialylated *N*-glycan stock were structurally analyzed via a multicapillary electrophoresis-based ABI3130 sequencer. Peaks were further analyzed with GeneMapper v3.7 software (Applied Biosystems, Foster City, CA, USA) and peak height intensities were normalized to the total intensity of the measured peaks. Different ratios and urinary glycoprofile markers (UGMs) were calculated as previously described [23,24,25].

### 2.3. Statistical Analysis

Statistical analyses were carried out using MedCalc v20.110 (MedCalc Software, Ostend, Belgium) and GraphPad Prism v8.0.2 (GraphPad Software Inc., La Jolla, CA, USA). The normal distribution of all continuous datasets (iPSA, sPSA density, and *N*-glycan ratios) was tested for normality using the D’Agostino-Pearson test. A comparison of non-parametric continuous variables (iPSA and sPSA density) between treatment cohorts was carried out using the 2-sided Kruskal–Wallis test with a post hoc Dunn test for further intergroup comparisons. Differences in non-parametric continuous variables (iPSA, sPSA density and *N*-glycan ratios) according to the occurrence of any event were assessed via the 2-sided Mann–Whitney U test. An event was classified as biochemical recurrence (RP/EBRT) according to EAU guidelines, the start of active (AS) or salvage therapy (RP/EBRT), increase in ISUP grade on biopsy (AS), clinical PD (AS/RP/EBRT), or death (AS/RP/EBRT), whichever occurred first. Significant differences in categorical variables were determined via Chi-square (*m* × *n* tables) and Fisher Exact testing (2 *×* 2 tables with less than ten observations for each cell). EFS after up to 10 years of follow-up was determined from the moment that therapy was started until the occurrence of an event. The hazard ratio (HR) was determined using a 2-sided log-rank (Mantel–Cox) test. Survival curves were plotted using the Kaplan–Meier method. Continuous prognostic scores were dichotomized for survival analysis by using the associated criterion for the highest Youden index. *p*-values < 0.05 were considered statistically significant. Taking the small number of patients into account, *p*-values between 0.05 and 0.10 were considered to indicate a trend toward significance.

## 3. Results

### 3.1. Patient Characteristics

The characteristics of the patients are shown in Table 1. The median iPSA concentrations were 8.8 ng/mL. The majority of patients presented with ISUP grade 1 (26%), ISUP grade 2 (35%), or ISUP grade 3 (21%). ISUP grade was significantly different between the treatment cohorts (*p* < 0.0001), with more indolent and aggressive cancers seen in the AS and EBRT cohorts, respectively. Next, patients mostly presented with T2 tumors (65%). In addition, T stage was positively associated with ISUP grade (*p* = 0.0091). Significantly more T1 tumors were seen in the AS cohort compared to the RP and EBRT cohort (*p* = 0.0037). Only a limited number of patients had lymph node invasion (4%), all of them being treated with EBRT. Positive surgical margins were observed in 27% of patients who underwent RP.

### 3.2. Assocation of Clinical and Biochemical Parameters to Occurence of Event

Twenty-six patients (34%) developed an event following primary therapy for PCa. The occurrence of an event was associated with higher T stage (*p* = 0.0195, Table 2) and higher ISUP grade (*p* = 0.0006, Table 2) but not N stage (*p* = 0.2621, Table 2). Only a trend toward significantly higher iPSA concentrations was noted in patients who eventually developed an event (median iPSA of 8.1 ng/mL vs. 13.2 ng/mL; *p* = 0.0538). Using the D’Amico Risk Classification for PCa evaluation, patients with the highest risk classification had an insignificant higher chance of developing any PD (*p* = 0.1833, Table 2).

**Figure 2:**
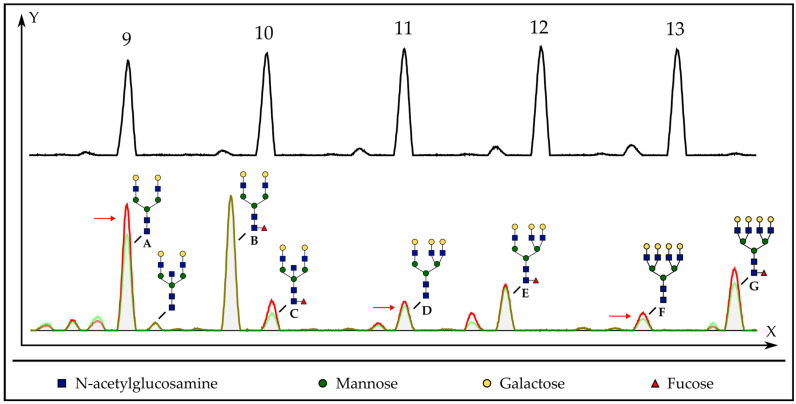
Comparison of urine *N*-glycome in patients with and without occurrence of an event. X-axis indicates time to elution; Y-axis demonstrates the relative number of fluorescence units. In the upper panel, the electropherogram of a dextran ladder is displayed. In the lower panel, the overlap of the median electropherogram of patients with and without an event is illustrated (red vs. green electropherogram). Identified glycosylation structures are shown next to their respective peak in the electropherogram. Glycan symbols are in accordance with the Consortium for Functional Glycomics (https://www.functionalglycomics.org/ (accessed on 28 November 2023)). The most important differences are indicated by a red arrow. Patients with an event following primary therapy for PCa showed lower 2AFc/2T ratios (=[peak B + C]/[peaks A + B + C]), 2AFc/MA ratios (=[peak B + C]/[sum of peaks A to G]) and Fc/MA ratios ([peak B + C]/[sum of peaks A to G]). 2AFc/2T, ratio of fucosylated biantennary structures/total biantennary structures; 2AFc/MA, ratio of fucosylated biantennary structures/total multiantennary structures; Fc/MA, ratio of fucosylated structures/total multiantennary structures; and PCa, prostate cancer.

**Figure 3:**
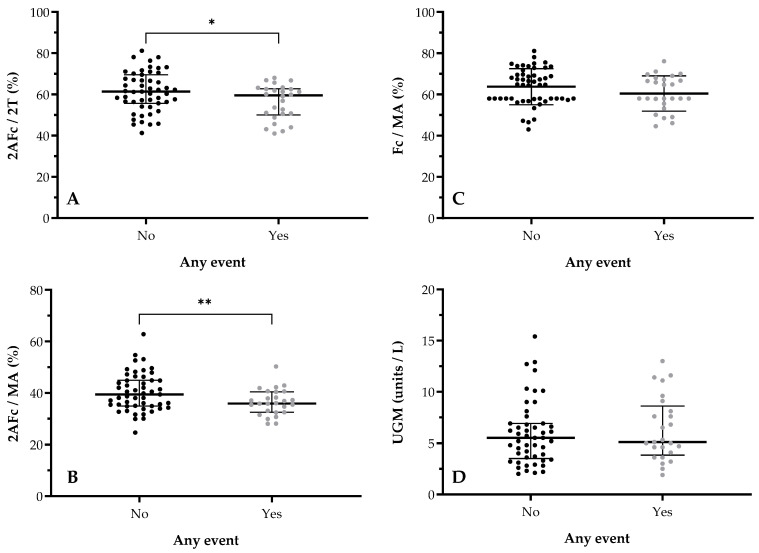
Differences in total urine *N*-glycome for occurrence of event. Y-axis depicts *N*-glycosylation ratio (%); X-axis depicts disease status (any event vs. no event). Data comparison graphs are depicted for (**A**) 2AFc/2T and occurrence of event (*p* = 0.0492), (**B**) 2AFc/MA and occurrence of event (*p* = 0.0253), (**C**) Fc/MA and occurrence of event (*p* = 0.1818), and (**D**) UGM and occurrence (*p* = 0.5876). Intergroup significant changes are indicated in the figure (* *p* < 0.05; ** *p* < 0.01). 2AFc/2T, ratio of fucosylated biantennary structures/total biantennary structures; 2AFc/MA, ratio of fucosylated biantennary structures/total multiantennary structures; Fc/MA, ratio of fucosylated structures/total multiantennary structures.

Next, total urine *N*-glycome was evaluated. The median urine *N*-glycosylation profile for patients with and without an event is shown in Figure 2. Patients without an event had significantly higher fucosylation ratios for fucosylated biantennary/total biantennary structures (2AFc/2T; median of 61.4% vs. 59.5%, *p* = 0.0492, Figure 3A) and for fucosylated biantennary/total multiantennary structures (2AFc/MA; median of 39.4% vs. 35.9%, *p* = 0.0253; Figure 3B) and an insignificantly higher total number of fucosylated structures/total multiantennary structures (Fc/MA; median of 64.7% vs. 58.0%, *p* = 0.1818; Figure 3C). No significance was observed for all other *N*-glycan ratios, including for the previously used diagnostic Urinary Glycoprofile Marker (median of 5.5 U/L vs. 5.1 U/L, *p* = 0.5876; Figure 3D). In addition, (borderline) higher 2AFc/2T (median of 61.3% vs. 55.3%, *p* = 0.0897) and Fc/MA ratios (median of 64.9% vs. 58.0%, *p* = 0.1027) were seen for ISUP grade 1–3 vs. ISUP grade 4–5, respectively.

### 3.3. EFS

The most optimal cut-off values for univariate EFS analysis per significant variable (iPSA, 2AFc/MA, Fc/MA, and 2AFc/2T) are shown in Table 3.

**Figure 4:**
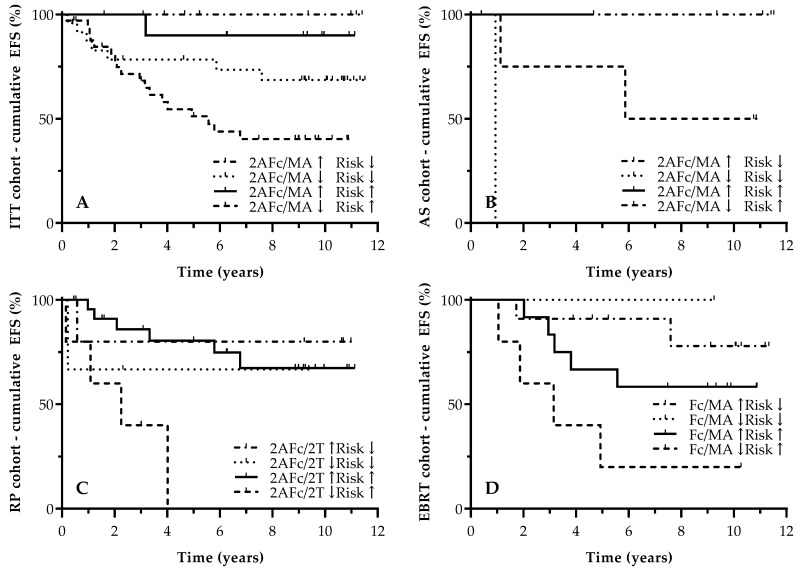
Kaplan–Meier EFS curves for combination of D’Amico Risk Classification for PCa and urine *N*-glycosylation ratios. The Y-axis depicts cumulative EFS (%); the X-axis depicts survival time in years. EFS curves are demonstrated for (**A**) ITT cohort (10-year EFS of 100.0%, 68.6%, 90.0%, and 40.2% for 2AFc/MA↑ Risk↓, 2AFc/MA↓ Risk↓, 2AFc/MA↑ Risk↑, and 2AFc/MA↓ Risk↑, respectively; *p* = 0.0057), (**B**) AS cohort (10-year EFS of 100.0%, 0.0%, 100.0% and 50.0% for 2AFc/MA↑ Risk↓, 2AFc/MA↓ Risk↓, 2AFc/MA↑ Risk↑, and 2AFc/MA↓ Risk↑, respectively; *p* = 0.0129), (**C**) RP cohort (10-year EFS of 80.0%, 66.7%, 67.3% and 0.0% for 2AFc/2T↑ Risk↓, 2AFc/2T↓ Risk↓, 2AFc/2T↑ Risk↑, and 2AFc/2T↓ Risk↑, respectively; *p* = 0.0198), and (**D**) EBRT cohort (10-year EFS of 77.9%, 100.0%, 58.3% and 20.0% for Fc/MA↑ Risk↓, Fc/MA↓ Risk↓, Fc/MA↑ Risk↑, and Fc/MA↓ Risk↑, respectively; *p* = 0.0413). Censored data are indicated in the graph. 2AFc/2T, ratio of fucosylated biantennary structures/total biantennary structures; 2AFc/MA, ratio of fucosylated biantennary structures/total multiantennary structures; AS, active surveillance; EBRT, external beam radiotherapy; Fc/MA, ratio of fucosylated structures on total of multiantennary structures; ITT, intention-to-treat; EFS, progression-free survival; and RP, radical prostatectomy.

Univariate survival analysis for all patients included (Table 4A) showed that low iPSA, low T stage, low ISUP grade and, by extension, having a low D’Amico Risk Classification for PCa, were associated with improved EFS (Figure S1A). In addition, several glycosylation ratios showed significant prognostic properties. Patients with high ratios of 2AFc/MA (>42.9%, HR = 0.29 [0.13–0.68], *p* = 0.0045), Fc/MA (>71.1%, HR = 0.36 [0.14–0.93], *p* = 0.0356), and 2AFc/2T (>66.9%, HR = 0.33 [0.13–0.80], *p* = 0.0144) demonstrated longer EFS in comparison to patients with ratios lower than the calculated cut-off values (Table 4A; Figures S2A, S3A and S4A). Combining the D’Amico Risk Classification for PCa together with the most significant *N*-glycan ratio (2AFc/MA) resulted in a tetra-categorical parameter with clear divergence in terms of EFS. In this model, patients with high 2AFc/MA ratios, irrespective of BCR Risk classification, demonstrated a 10-year EFS of 90.0% to 100.0% (*p* = 0.0057, Figure 4A).

Further subanalysis per treatment group resulted in more or less similar results. In the AS cohort, apart from iPSA, none of the other clinical parameters (T stage or ISUP grade and, as a consequence, the combined D’Amico Risk Classification for PCa) reached statistical significance (Table 4B, Figure S1B). Comparable to the ITT cohort, having higher ratios of 2AFc/MA, Fc/MA, and 2AFc/2T resulted in a prolonged EFS; however, this benefit was only significant for 2AFc/MA (Table 4B, Figures S2B, S3B and S4B). The construction of a model that included the D’Amico Risk Classification for PCa and the 2AFc/MA ratio yielded significant EFS results for combinations including high 2AFc/MA ratios, with no event after 10 years of follow-up (*p* = 0.0129, Figure 4B).

For the RP cohort, iPSA and T stage proved to be associated with EFS. Surprisingly, neither ISUP grade nor the presence of positive surgical margins nor the D’Amico Risk Classification for PCa were associated with EFS following RP (Table 4C, Figure S1C). With respect to the *N*-glycan biomarker, having both a higher 2AFc/MA ratio (>40.7%, HR = 0.27 [0.08–0.85], *p* = 0.0260) and higher 2Afc/2T ratio (>52.7%, HR = 0.12 [0.02–0.60], *p* = 0.0097) was linked to prolonged EFS (Figures S2C, S3C, and S4C). Combining the 2AFc/2T ratio with the D’Amico Risk Classification for PCa resulted in a significant EFS model, with all patients in the less favorable group having suffered an event after 10 years of follow-up (*p* = 0.0198, Figure 4C).

Lastly, in the EBRT cohort, all of the clinical parameters (iPSA, T stage, ISUP grade) demonstrated a significant association to EFS; however, the D’Amico Risk Classification for PCa only showed a trend towards significance (Figure S1D). No *N*-glycan ratio resulted in any significant EFS outcome; however, a trend towards a significance was found for Fc/MA (>49.0%, HR = 0.21 [0.04–1.06], *p* = 0.0589, Table 4D, Figures S2D, S3D, and S4D). Combining the latter *N*-glycan parameter with the D’Amico Risk Classification for PCa resulted in a significant model for EFS following the administration of EBRT with a 10-year EFS of 77.9% to 100.0% in the most favorable subgroups (*p* = 0.0413, Figure 4D).

## 4. Discussion

The objective of this study was to evaluate the prognostic properties of urine *N*-glycome, measured at diagnosis, in PCa patients who received primary therapy (RP and EBRT) or were actively surveyed for their disease and followed up for up to 12 years.

With regard to the clinical parameters, our patient population is in line with the data reported in the literature. The youngest patients were seen in the RP cohort. This is logical as RP is less suitable for frail and or unfit patients [26]. Next, iPSA gradually increased from the AS cohort to the RP cohort and the EBRT cohort.

ISUP grade and T stage and, consequently, the D’Amico risk groups were higher in patients undergoing RP or receiving EBRT vs. patients followed up with AS. This is again in concordance with the literature as the guidelines stipulate monitoring indolent cancers using AS, whereas patients with more aggressive tumors need to be referred for more active therapy, such as RP and EBRT [2]. This also holds true for our ITT cohort, as almost all T3–4 tumors and N+ tumors were treated using RP or EBRT.

In our study, with a median follow-up of 9.4 years, PD was observed in 34% of patients. Logically, PD occurred mostly in patients with high ISUP grades (ISUP grade 4–5) and in patients with extraprostatic extension of their PCa (T3–4 stages). Focusing on each separate treatment arm, we noted that the rates of PD were 27%, 32%, and 38% in the AS, RP, and EBRT cohorts, respectively. The event rate of PD is in concordance with the known literature. A meta-analysis conducted by Rajwa et al. [13] indicated a pooled PCa event rate of 27% in patients undergoing AS, which is similar to the event rate observed in our study [13]. Next, the data from the large EAU database indicate a 27% PD rate following radical prostatectomy [27]. Lastly, a review undertaken by Artibani et al. [14] reported findings of BCR in 27% to 53% of patients receiving EBRT, which is consistent with our observations [14].

Urine *N*-glycome was linked to the occurrence of PD. It was found that core-fucosylation plays a pivotal role in the progression of PCa. Here, more specifically, the core-fucosylation of biantennary *N*-glycans was lower in patients who eventually developed PD. Furthermore, core-fucosylation ratios were also lower in patients with ISUP grade ≥ 4. This finding is in agreement with the observations of our initial diagnostic study, in which the 2AFc/2T and Fc/MA ratios were also significantly lower in patients with Gleason scores of ≥8 [23]. In addition, other research groups have also illustrated the association between low core-fucosylation ratios, specifically on the almost exclusively biantennary glycosylated PSA, and more aggressive disease status [28,29].

The 5-year EFS in our study was 90.0%, 70.0%, and 68.6% for AS, RP, and EBRT, deviating slightly from the EFS indicated in the literature. This is probably attributable to the lower number of patients in each study arm [30,31,32]. As expected, all separate clinical parameters (iPSA, T stage, and Gleason score), as well as the combined risk nomogram (D’Amico risk classification), successfully prognosticated PCa patients for EFS in the ITT cohort.

During subanalysis, it was surprising to see that the inferior clinical parameters (having high T stage and ISUP grades and, subsequently, poor D’Amico risk classification) were not associated with a significant worsening of EFS in the AS cohort. The most logical explanation for this observation is the lower number of patients (*n* = 11) in the AS cohort. This is especially true as the longest EFS was observed for the most favorable groups (with an iPSA below the calculated threshold of 12.0 ng/mL, T1 tumors, and favorable D’Amico risk classification).

For RP, almost every clinical parameter showed significance for EFS. Increasing iPSA concentrations were linked to shorter EFS. This finding is consistent with the statement by Van den Broeck et al. [33] that iPSA is an important preoperative predictor for BCR [33]. Additionally, prognostication using T stage yielded a significant outcome, as the performance in cases of T3 tumors was worse in this cohort. Such an observation has also been highlighted in a review performed by Van den Broeck et al. [33], although the prognostic use of T stage is controversial. The overall ISUP grade had no effect on EFS outcome in the RP cohort, contradicting the consensus regarding the negative prognostic value of increasing ISUP scores for oncological outcomes [33,34]. As a bicategorical ISUP grade (ISUP grade ≤ 3 vs. ISUP grade ≥ 4) resulted in a significant difference with regard to EFS in our study, the lower number of patients will probably account for the non-significant finding.

Lastly, all of the clinical parameters showed significant prognostic properties with regard to EFS in the EBRT cohort. Of note, patients diagnosed with ISUP grade 3 yielded higher EFS results compared to patients with ISUP grade 2.

Similar to the occurrence of PD, the 2AFc/2T, 2AFc/MA and Fc/MA ratios were significantly associated with EFS outcomes, with lower core-fucosylation rates being linked to worse EFS. On the one hand, this finding can again be linked to the observation that prostate proteins derived from more aggressive PCa demonstrate a lower core-fucosylation content in their carbohydrate structures [23]. The construction of a nomogram based on the D’Amico risk classification and urine *N*-glycan markers resulted in tetracategorical classification, which further subdivided patients according to their risk for PD. In this nomogram, it was clear that patients with a low D’Amico risk classification and high core-fucosylation content have the most favorable EFS, with a 10-year EFS of 100%. Otherwise, patients included in the highest risk only had a 10-year EFS of 40.2%. Therefore, the nomogram provides substantial information with regard to monitoring and follow-up intensification.

Furthermore, it was interesting to notice that different *N*-glycan ratios were associated with EFS outcomes in the subgroup analysis. In AS, 2AFc/MA was found to be the most significant *N*-glycome ratio for prognosis. Secondly, in the RP cohort, both the 2AFc/2T and 2AFc/MA ratios showed prognostic properties in relation to EFS. The observation for the latter ratio is in concordance with the findings for tissue *N*-glycosylation, in which the 2AFc/MA ratio, among other *N*-glycan patterns, was significantly associated with BCR-free survival in PCa patients undergoing RP [22]. On the other hand, in the EBRT cohort, only the overall Fc/MA ratio showed a trend toward significant association to EFS. It can be assumed that this difference in applied prognostic *N*-glycan markers can be attributed to the fact that the majority of patients with the most aggressive local/localized PCa were included in our EBRT cohort. As it has been illustrated that *N*-glycan patterns differ significantly according to disease status, it is logical that other *N*-glycan ratios will be applied to correct prognostication regarding EFS in AS, RP, and EBRT cohorts.

It does remain interesting to see that only high fucosylation ratios in urine were linked to improved EFS after primary therapy in PCa patients. This does not concur with the statement from Scott and Munkley, who reported that increased α-1,6-core-fucosylation is associated with PD [35]. Additionally, Wang et al. indicated that α-1,6-fucosylation is increased in high-grade and metastatic PCa [36]. The discordance between studies may be related to the type of specimen analyzed, as the carbohydrate structure has a pivotal role in the secretion and transport of glycoproteins [37].

Next, there is accumulating evidence suggesting increased β-1,6-branch production via *N*-acetylglucosaminyltransferase-V during cancer progression [38]. Here, we did not find β-1,6-branched *N*-glycan structures to be related to reduced EFS. This can again be explained by the type of specimen analyzed or the fact that an increase in β-1,6-branching is a feature that develops further on during cancer progression, resulting in a less pronounced effect during diagnosis. A third possibility is the localization of the tumor in the prostate gland. It is assumable that DRE of the prostate with a tumor being more distant from the urethra yields fewer tumoral proteins in comparison to a tumor that is closer to the urethra, thus resulting in decreased effectiveness when measuring tumoral *N*-glycan patterns. However, the fact that we did notice differences in the fucosylation ratios with regard to EFS makes this argument less plausible.

Irrespective of the aforementioned hypotheses, it remains clear that additional research is needed to study tumor glycobiology in depth in an attempt to explain why certain glycosylation changes occur in PCa and why these changes are linked to specific body fluids examined (blood vs. urine). For liver proteins, it has been stated that proteins with core fucose are mostly secreted on the apical side (bile duct side) [39]. The same may be true for PCa, where urine and blood are at the respective apical and basolateral sides. Contrary to the normally expected increase in fucose in urine, a breakdown of cancer cell polarity and a subsequent reduction in urinary core-fucosylated proteins may point toward more aggressive disease and poorer outcomes [40].

Our study has some limitations. Firstly, there was a limited number of patients (*n* = 77) included in this study, which did, due to the consequently low number of events per treatment group, not allow for a more in-depth multivariate EFS analysis. Nevertheless, post hoc analysis indicated a power of over 90% in our study based on the most potent *N*-glycan parameters for the ITT cohort (two-tailed, *p* < 0.05, 10-year event-free survival proportion 2AFc/MA _LOW_ = 0.52, 10-year event-free survival proportion 2AFc/MA _HIGH_ = 0.94, N 2AFc/MA _LOW_ = 58, N 2AFc/MA _HIGH_ = 19). The significant results in our small cohort urge the need for a larger validation study to determine the full prognostic value of urine and blood N-glycan biomarkers in relation to PCa.

Secondly, we only evaluated urine collected at the time of diagnosis. Therefore, we have no continuous follow-up of the urine *N*-glycan profile after the start of therapy, which might yield additional important information. However, the fact that the urine *N*-glycan profile evaluated at diagnosis can prognosticate patients following primary therapy for PCa can be perceived as one of the major advantages of the study, as patients could be more accurately managed in a curative setting.

Thirdly, we focused on the total urine *N*-glycome of all prostate proteins present. Further in-depth analysis of PSA derived from the urine samples may even improve the prognostic value of urine *N*-glycosylation biomarkers. This is especially relevant as PSA is mostly biglycosylated and as we have mostly noted differences in the fucosylation of biantennary structures in our current study. In addition, we specifically focused on the most abundantly present desialylated carbohydrate structures. Evaluating the sialylation status of urinary *N*-glycans, as well as less abundant glycoforms, may provide additional insights into tumor glycobiology and potentially prove to have prognostic value.

## 5. Conclusions

We have illustrated that urine *N*-glycan core-fucosylation ratios, evaluated at diagnosis of the PCa, show potential as a prognostic tool for EFS following primary PCa therapy. Combining urine *N*-glycan biomarkers with the D’Amico Risk Classification for PCa resulted in improved patient prognostication. Further prospective evaluation is needed to allow for urine *N*-glycan biomarkers to be incorporated into daily clinical practice to optimize patient guidance after primary PCa therapy.

## Figures and Tables

**Table 1:** Patient characteristics.

Parameter		ITT Cohort	AS	RP	EBRT
N		77 (100)	11 (14)	37 (48)	29 (38)
Age initial diagnosis, years		66 (50–79)	62 (50–79)	64 (52–77)	68 (52–78)
iPSA, ng/mL *		8.8 (1.0–71.0)	7.9 (1.0–18.0)	8.1 (1.8–36.0)	12.9 (2.2–71.0)
sPSA density, ng/mL² *		0.238 (0.033–2.075)	0.151 (0.033–0.375)	0.223 (0.048–1.199)	0.272 (0.100–2.075)
ISUP grade ^§^	1	20 (26)	10 (91)	6 (16)	4 (14)
	2	27 (35)	1 (9)	18 (49)	8 (28)
	3	16 (21)	0 (0)	8 (22)	8 (28)
	4	5 (6)	0 (0)	3 (8)	2 (6)
	5	9 (12)	0 (0)	2 (5)	7 (24)
T ^§^	1a	1 (1)	1 (9)	0 (0)	0 (0)
	1b	1 (1)	0 (0)	0 (0)	1 (3)
	1c	9 (12)	4 (36)	0 (0)	5 (17)
	2a	17 (22)	4 (36)	6 (16)	7 (24)
	2b	7 (9)	2 (18)	3 (8)	2 (7)
	2c	26(34)	0 (0)	18 (49)	8 (28)
	3a	13 (17)	0 (0)	10 (27)	3 (10)
	3b	2 (3)	0 (0)	0 (0)	2 (7)
	4	1 (1)	0 (0)	0 (0)	1 (3)
N ^§^	0	74 (96)	11 (100)	37 (100)	26 (90)
	1	3 (4)	0 (0)	0 (0)	3 (10)
D’Amico Risk Classification for PCa	Low	8 (10)	5 (45)	1 (3)	2 (7)
	Intermediate	23 (30)	6 (55)	7 (19)	10 (34)
	High	46 (60)	0 (0)	29 (78)	17 (59)
Therapy	AS	11 (14)	11 (100)	0 (0)	0 (0)
	RP	7 (9)	0 (0)	7 (19)	0 (0)
	RP + PLND	30 (39)	0 (0)	30 (81)	0 (0)
	EBRT	5 (6)	0 (0)	0 (0)	5 (17)
	EBRT + ADT	5 (6)	0 (0)	0 (0)	5 (17)
	EBRT +PLND + ADT	19 (25)	0 (0)	0 (0)	19 (66)
R	Positive	- (-)	- (-)	10 (27)	- (-)
	Negative	- (-)	- (-)	27 (73)	- (-)
Duration follow-up, years		9.4 (9.1–10.1)	10.9 (4.7–11.4)	9.2 (6.3–9.6)	9.9 (9.0–10.3)
PD	No	51 (66)	8 (73)	25 (68)	18 (62)
	BCR	10 (13)	0 (0)	8 (22)	2 (7)
	Increased ISUP grade	3 (4)	3 (27)	0 (0)	0 (0)
	Increased T stage	1 (1)	0 (0)	0 (0)	1 (3)
	M+ disease	2 (3)	0 (0)	1 (3)	1 (3)
	Deceased	10 (13)	0 (0)	3 (8)	7 (24)

All data are *n* (%) except for age at initial diagnosis, iPSA, sPSA density: median (range), and for the duration of follow-up: median (95% CI). * iPSA and sPSA density were significantly different between the AS and EBRT cohorts (*p* = 0.0299 and *p* = 0.0059, respectively). ^§^ ISUP grade and T stage were significantly different between the treatment cohorts (*p* < 0.0001 and *p* = 0.0037, respectively). T and N stage were determined using pathological assessment (RP), MRI (if available), or through clinical evaluation (digital rectal examination) (latter only applicable for T stage). N+ disease was exclusively seen in the EBRT cohort but was only trending to be significantly different from the other cohorts (*p* = 0.0755). The D’Amico Risk Classification for PCa is based on the combination of iPSA, T stage, and ISUP grade. ADT, androgen deprivation therapy; AS, active surveillance; BCR, biochemical recurrence; CI, confidence interval; EBRT, external beam radiotherapy; iPSA, initial serum prostate-specific antigen; ISUP, International Society of Urological Pathology; MRI, magnetic resonance imaging; NR, not reached; PCa, prostate cancer; PD, progressive disease; PLND, pelvic lymph node dissection; R, resection margins; RP, radical prostatectomy; and sPSA, serum prostate-specific antigen.

**Table 2:** Association between clinical parameters and the occurrence of an event.

Parameter		Any Event		*p* Value
		No (*n* = 51)	Yes (*n* = 26)	
ISUP grade	1	16 (80)	4 (20)	0.0006
	2	18 (67)	9 (33)	
	3	14 (88)	2 (13)	
	4–5	3 (21)	11 (79)	
T	1	10 (91)	1 (9)	0.0195
	2	35 (70)	15 (30)	
	3	6 (40)	9 (60)	
	4	0 (0)	1 (100)	
N	0	50 (98)	1 (2)	0.2621
	1	24 (92)	2 (8)	
D’Amico Risk Classification for PCa	Low	7 (14)	1 (4)	0.1833
	Intermediate	17 (33)	6 (23)	
	High	27 (53)	19 (73)	

All data are *n* (%). The D’Amico Risk Classification for PCa is based on the combination of iPSA, T stage, and ISUP grade. Significant results are highlighted in green. BCR, biochemical recurrence; iPSA, initial serum prostate-specific antigen; ISUP, International Society of Urological Pathology; and PCa, prostate cancer.

**Table 3:** Calculated cut-off values for EFS.

Parameter	ITT Cohort (*n* = 77)	AS Cohort (*n* = 11)	RP Cohort (*n* = 36)	EBRT Cohort (*n* = 29)
iPSA	>13.6 ng/mL	>12.0 ng/mL	>8.4 ng/mL	>15.8 ng/mL
2AFc/MA	>42.9%	>33.1%	>40.7%	>37.9%
Fc/MA	>71.1%	>66.7%	>64.9%	>49.0%
2AFc/2T	>66.9%	>66.9%	>52.7%	>68.0%

2AFc/2T, ratio of fucosylated biantennary/total biantennary structures; 2AFc/MA, ratio of fucosylated biantennary structures/total multiantennary structures; AS, active surveillance; EBRT, external beam radiotherapy; EFS, event-free survival; Fc/MA, ratio of total number of fucosylated structures/total multiantennary structures; iPSA, initial serum prostate-specific antigen; ITT, intention-to-treat; ROC, receiver operating curve; and RP, radical prostatectomy.

**Table 4:** Univariate proportional hazard model for EFS.

	Parameter		Mean EFS (Years)	HR (95% CI)	*p* Value
**A.** **ITT cohort**	iPSA	≤13.6 ng/mL	9.2	1	0.0067
		>13.6 ng/mL	6.1	3.35 (1.40–8.01)	
	T stage	1	10.6	1	0.0008
		2	8.7	3.83 (1.36–10.7)	
		3–4	4.3	12.3 (3.12–46.8)	
	ISUP grade	1	9.4	1	0.0005
		2	8.4	1.58 (0.59–4.19)	
		3	9.6	0.62 (0.20–1.88)	
		4–5	4.5	4.97 (1.42–17.4)	
	D’Amico Risk Classification for PCa	Low–intermediate	9.4	1	0.0874
		High	7.3	1.97 (0.91–4.29)	
	2AFc/MA	≤42.9%	7.4	1	0.0039
		>42.9%	10.9	0.29 (0.12–0.67)	
	Fc/MA	≤71.1%	7.8	1	0.0356
		>71.1%	10.9	0.36 (0.14–0.93)	
	2AFc/2T	≤66.9%	7.6	1	0.0144
		>66.9%	10.8	0.33 (0.13–0.80)	
**B.** **AS cohort**	iPSA	≤12.0 ng/mL	10.2	1	0.0335
		>12.0 ng/mL	3.5	23.4 (1.28–426)	
	T stage	1	9.4	1	0.5740
		2	8.2	1.92 (0.20–18.7)	
	ISUP grade	1	8.5		0.5258
		2	10.9		
	D’Amico Risk Classification for PCa	Low	9.4	1	0.5492
		Intermediate	7.6	2.00 (0.21–19.5)	
	2AFc/MA	≤33.1%	10.2	1	0.0123
		>33.1%	3.4	0.02 (0.00–0.41)	
	Fc/MA	≤66.7%	7.3	1	0.3578
		>66.7%	10.3	0.33 (0.03–3.47)	
	2AFc/2T	≤66.9%	11.4		0.0979
		>66.9%	6.8		
**C.** **RP cohort**	iPSA	≤8.4 ng/mL	9.1	1	0.0783
		>8.4 ng/mL	6.4	2.81 (0.89–8.88)	
	T stage	2	8.7	1	0.0359
		3	4.1	4.87 (1.11–21.4)	
	ISUP grade	1	9.0	1	0.6445
		2	8.1	1.73 (0.33–9.12)	
		3	8.1	1.50 (0.22–10.4)	
		4–5	5.4	3.37 (0.40–28.4)	
	D’Amico Risk Classification for PCa	Low–intermediate	8.3	1	0.6066
		High	7.7	1.43 (0.37–5.51)	
	Positive surgical margins	No	8.0	1	0.6991
		Yes	7.3	1.32 (0.32–5.40)	
	2AFc/MA	≤40.7%	10.3	1	0.0260
		>40.7%	6.5	0.27 (0.08–0.85)	
	Fc/MA	≤64.9%	9.4	1	0.1045
		>64.9%	6.7	0.39 (0.12–1.22)	
	2AFc/2T	≤52.7%	8.8	1	0.0097
		>52.7%	3.8	0.12 (0.02–0.60)	
**D.** **RT cohort**	iPSA	≤15.8 ng/mL	9.5	1	0.0311
		>15.8 ng/mL	6.0	4.03 (1.14–14.3)	
	T stage	1	11.3		0.0132
		2	8.3		
		3–4	4.5		
	ISUP grade	1	11.3		0.0001
		2	8.1		
		3	10.9		
		4–5	3.8		
	D’Amico Risk Classification for PCa	Low–intermediate	10.1	1	0.0010
		High	5.1	9.34 (2.48–35.2)	
	2AFc/MA	≤37.9%	9.9	1	0.1168
		>37.9%	7.1	0.38 (0.11–1.27)	
	Fc/MA	≤49.0%	9.0	1	0.0589
		>49.0%	5.3	0.21 (0.04–1.06)	
	2AFc/2T	≤68.0%	9.9		0.1000
		>68.0%	7.7		

Significant results are highlighted in green; the trend toward significance is highlighted in orange. 2AFc/2T, ratio of fucosylated biantennary/total biantennary structures; 2AFc/MA, ratio of fucosylated biantennary structures/total multiantennary structures; AS, active surveillance; EBRT, external beam radiotherapy; EFS, event-free survival; Fc/MA, ratio of total number of fucosylated structures/total multiantennary structures; iPSA, initial serum prostate-specific antigen; ITT, intention-to-treat; ROC, receiver operating curve; and RP, radical prostatectomy.

## Data Availability

The data presented in this study are available upon request from the corresponding author (not publicly available due to European General Data Protection Regulation).

## Supplementary Materials

The following supporting information can be downloaded at: https://www.mdpi.com/article/10.3390/biomedicines12051039/s1, Figure S1: Kaplan-Meier EFS curves for D’Amico Risk Classification for PCa; Figure S2: Kaplan-Meier EFS curves for 2AFc/MA; Figure S3: Kaplan-Meier EFS curves for Fc/MA; Figure S4: Kaplan-Meier EFS curves for 2AFc/2T.
