# Supplementary material for: The Association between Urine N-Glycome and Prognosis after Initial Therapy for Primary Prostate Cancer
Source: Biomedicines. 2024 May 8;12(5):1039. doi: 10.3390/biomedicines12051039 (PMC11118943; doi:10.3390/biomedicines12051039)
Supplement: Supplementary file 1 [file biomedicines-12-01039-s001.zip › biomedicines-2924828-supplementary.pdf]

biomedicines

Article – supplementary materials

The Association between Urine *N*-Glycome and Prognosis after Initial Therapy for Primary Prostate Cancer

1. Supplementary figures

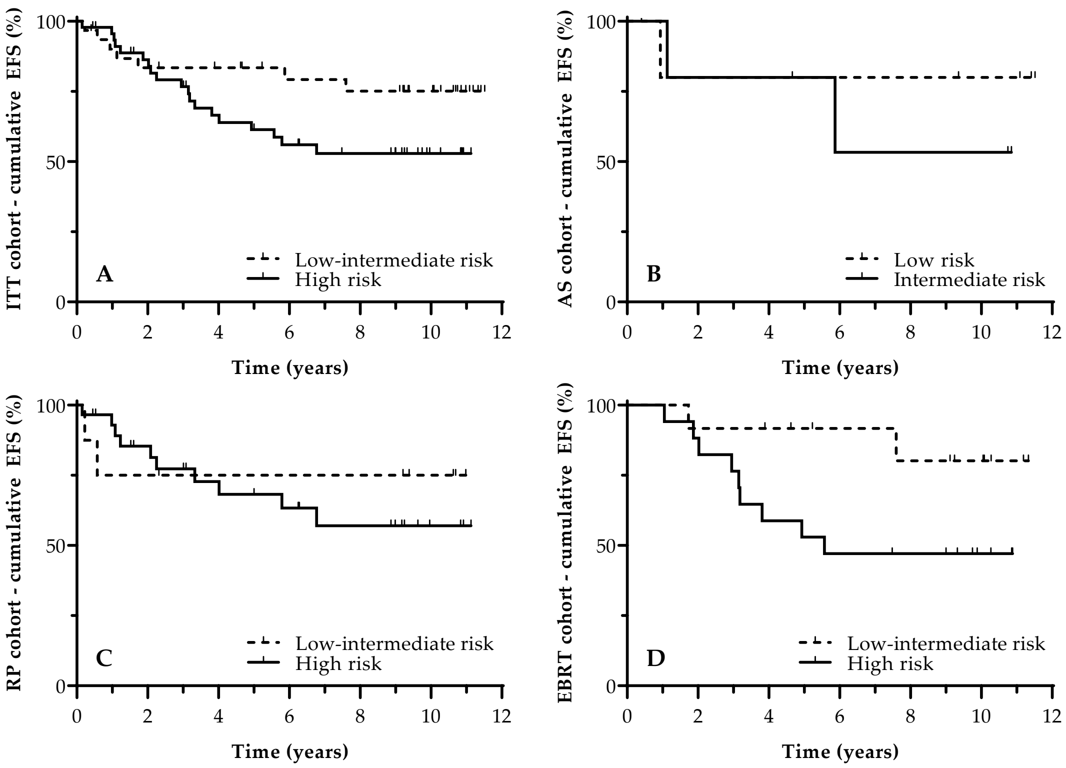

Figure S1. Kaplan-Meier EFS curves for D'Amico Risk Classification for PCa. Y-axis depicts cumulative EFS (%), X-axis depicts survival time in years. EFS curves are demonstrated for (A) ITT cohort ($HR_{high\ risk}$= 1.97 [0.91-4.29]; P = 0.0874), (B) AS cohort ($HR_{intermediate\ risk}$= 2.00 [0.21-19.5]; P = 0.5492), (C) RP cohort ($HR_{high\ risk}$= 1.43 [0.37-5.51]; P = 0.6066), and (D) EBRT cohort ($HR_{high\ risk}$= 9.34 [2.48-35.2]; P = 0.0010). Censored data are indicated in the graph. AS, active surveillance; EBRT, external beam radiotherapy; ITT, intention-to-treat; EFS, progression-free survival; RP, radical prostatectomy.

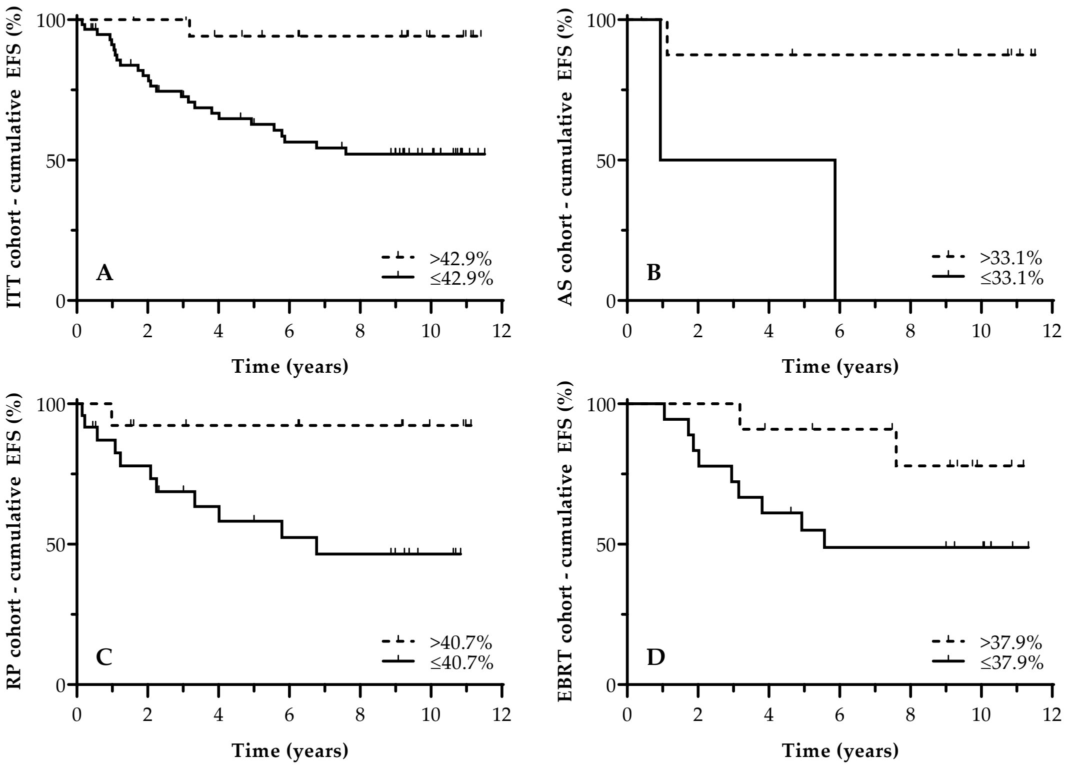

Figure S2. Kaplan-Meier EFS curves for 2AFc/MA. Y-axis depicts cumulative EFS (%), X-axis depicts survival time in years. EFS curves are demonstrated for (A) ITT cohort ($HR_{>42.9}$= 0.29 [0.12-0.67]; P = 0.0039), (B) AS cohort ($HR_{>33.1}$= 0.02 [0.00-0.41]; P = 0.0123), (C) RP cohort ($HR_{>40.7}$= 0.27 [0.08-0.85]; P = 0.0260), and (D) EBRT cohort ($HR_{>37.9}$= 0.38 [0.11-1.27]; P = 0.1168). Censored data are indicated in the graph. 2AFc/MA, ratio of fucosylated biantennary structures / total of multiantennary structures; AS, active surveillance; EBRT, external beam radiotherapy; ITT, intention-to-treat; EFS, progression-free survival; RP, radical prostatectomy.

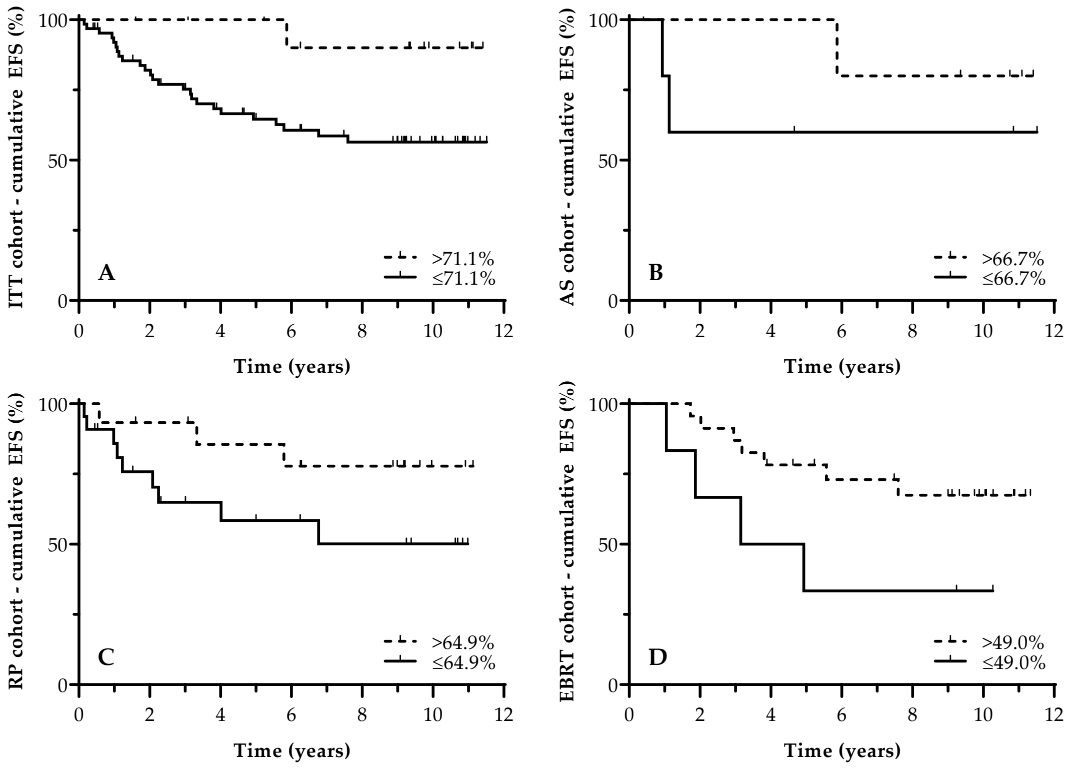

Figure S3. Kaplan-Meier EFS curves for Fc/MA. Y-axis depicts cumulative EFS (%), X-axis depicts survival time in years. EFS curves are demonstrated for (A) ITT cohort ($HR_{>71.1}$= 0.36 [0.14-0.93]; P = 0.0356), (B) AS cohort ($HR_{>66.7}$= 0.33 [0.03-3.47]; P = 0.3578), (C) RP cohort ($HR_{>64.9}$= 0.39 [0.12-1.22]; P = 0.1045), and (D) EBRT cohort ($HR_{>49.0}$= 0.21 [0.04-1.06]; P = 0.0589). Censored data are indicated in the graph. AS, active surveillance; EBRT, external beam radiotherapy; Fc/MA, ratio of fucosylated structures / total of multiantennary structures; ITT, intention-to-treat; EFS, progression-free survival; RP, radical prostatectomy.

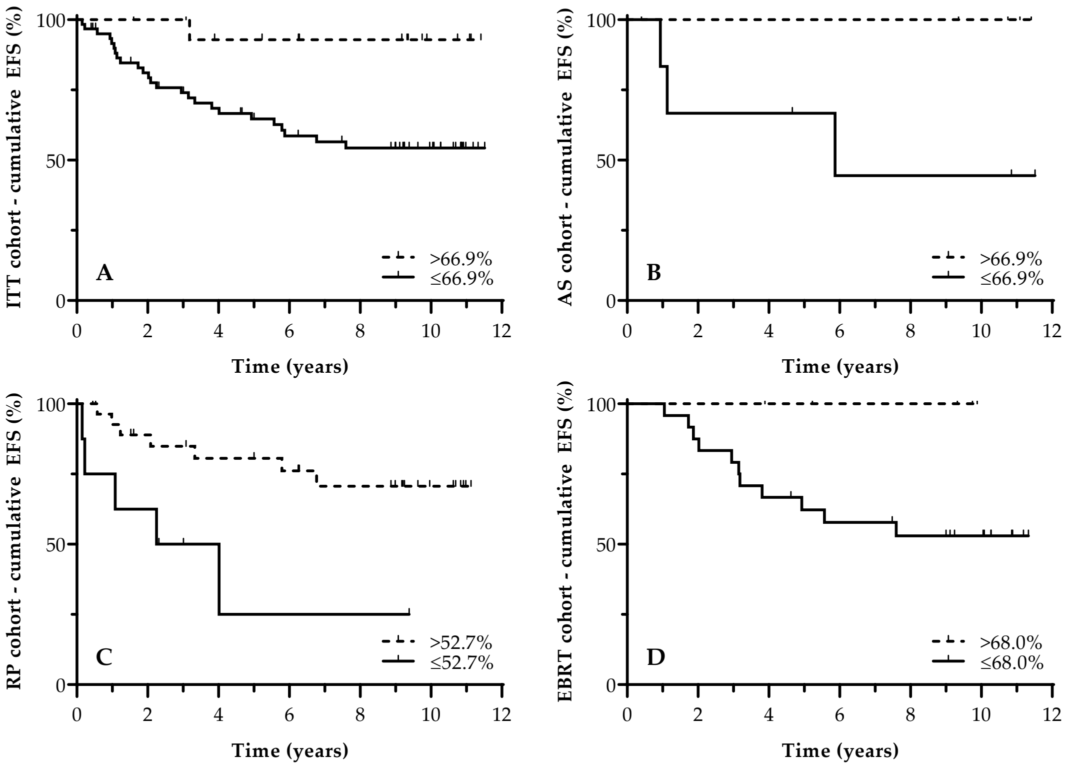

Figure S4. Kaplan-Meier EFS curves for 2AFc/2T. Y-axis depicts cumulative EFS (%), X-axis depicts survival time in years. EFS curves are demonstrated for (A) ITT cohort ($HR_{>66.9}$= 0.33 [0.13-0.80]; P = 0.0144), (B) AS cohort (P = 0.0979), (C) RP cohort ($HR_{>52.7}$= 0.12 [0.02-0.60]; P = 0.0097), and (D) EBRT cohort (P = 0.1000). Censored data are indicated in the graph. 2AFc/2T, ratio of fucosylated biantennary structures / total of biantennary structures; AS, active surveillance; EBRT, external beam radiotherapy; ITT, intention-to-treat; EFS, progression-free survival; RP, radical prostatectomy.
